# Fat Soluble Vitamins in Institutionalized Elderly and the Effect of Exercise, Nutrition and Cognitive Training on Their Status—The Vienna Active Aging Study (VAAS): A Randomized Controlled Trial

**DOI:** 10.3390/nu11061333

**Published:** 2019-06-14

**Authors:** Bernhard Franzke, Barbara Schober-Halper, Marlene Hofmann, Stefan Oesen, Anela Tosevska, Eva-Maria Strasser, Rodrig Marculescu, Barbara Wessner, Karl-Heinz Wagner

**Affiliations:** 1 Research Platform Active Ageing, University of Vienna, Althanstraße 14, 1090 Vienna, Austria; bernhard.franzke@univie.ac.at (B.F.); barbara.halper@univie.ac.at (B.S.-H.); marlene.hofmann@univie.ac.at (M.H.); stefan.oesen@gmail.com (S.O.); atosevska@ucla.edu (A.T.); barbara.wessner@univie.ac.at (B.W.); 2 Centre for Sport Science and University Sports, Department of Sports Medicine, Exercise Physiology and Prevention, University of Vienna, Auf der Schmelz 6, 1150 Vienna, Austria; 3 Department of Molecular, Cell and Developmental Biology, UCLA, 610 Charles E. Young Drive East, Los Angeles, CA 90095, USA; 4 Karl Landsteiner Institute for Remobilization and Functional Health/Institute for Physical Medicine and Rehabilitation, Kaiser Franz Joseph Spital, SMZ-Süd, Kundratstraße 3, 1100 Vienna, Austria; Eva-maria.strasser@wienkav.at; 5 Department of Laboratory Medicine, Division of Medical-Chemical Laboratory Diagnostics, Medical University of Vienna, Spitalgasse 23, 1090 Vienna, Austria; rodrig.marculescu@meduniwien.ac.at; 6 Department of Nutritional Sciences, Faculty of Life Sciences, University of Vienna, Althanstraße 14, 1090 Vienna, Austria

**Keywords:** aging, micronutrients, carotenoids, tocopherols, vitamin D, resistance training

## Abstract

Background: Institutionalized elderly are at higher risk for micronutrient deficiency. In particular, fat soluble micronutrients, which additionally have antioxidative function, are of interest. The purpose of this secondary investigation of the Vienna Active Ageing Study was to assess and evaluate the plasma status of retinol, alpha- and gamma-tocopherol, alpha- and beta-carotene, lutein, zeaxanthin, beta-cryptoxanthin, and lycopene, as well as vitamin D (25(OH)D) in a cohort of institutionalized elderly. We further determined the effect of six months strength training with or without supplementing (antioxidant) vitamins and protein on the plasma status of these ten micronutrients. Methods: Three groups (*n* = 117, age = 83.1 ± 6.1 years)—resistance training (RT), RT combined with protein and vitamin supplementation (RTS), or cognitive training (CT)—performed two guided training sessions per week for six months. Micronutrients were measured with High Performance Liquid Chromatography (HPLC) at baseline and after 6 months of intervention. Physical fitness was assessed by the 6-min-walking, the 30-s chair rise, isokinetic dynamometry, and the handgrip strength tests. Results: At baseline, the plasma status of retinol was satisfactory, for alpha-tocopherol, beta-carotene, and 25(OH)D, the percentage of individuals with an insufficient status was 33%, 73% and 61%/81% (when using 50 nmol/L or 75 nmol/L as threshold levels for 25(OH)D), respectively. Plasma analyses were supported by intake data. Six months of elastic band resistance training with or without protein-vitamin supplementation had no biological impact on the status of fat soluble micronutrients. Even for vitamin D, which was part of the nutritional supplement (additional 20 µg/d), the plasma status did not increase significantly, however it contributed to a lower percentage of elderly below the threshold levels of 50/75 nmol/L (49%/74%). Conclusions: The findings of the study lead to the strong recommendation for regular physical activity and increased consumption of plant-based foods in institutionalized elderly. When supported by blood analysis, supplementing micronutrients in a moderate range should also be considered.

## 1. Introduction

Increasing life-expectancy and consequently the incidence of age-related diseases is one main challenge for health systems. Sarcopenia, the age-related loss of muscle mass and function, as well as the aging process itself, are strongly linked to increased oxidative damage, which in turn is linked to age-related diseases [1]. In order to maintain an oxidative balance during the aging process, the plasma status of compounds with antioxidative potential is of high importance.

Elderly people are often at risk for inadequate nutrient supply due to physiological, metabolic, and age-associated factors affecting nutrient and fluid intake [2,3]. Chronic diseases, impaired digestion and absorption from the gastrointestinal tract, mal-/hypofunction of organs important for micronutrient metabolism, or nutrient-drug interactions, are prevalent in this population and can further compromise the nutritional status [4,5,6].

The prevalence of an inadequate nutritional status and of risk for malnutrition in older adults in Europe and North America ranges up to 85% depending on whether participants comprise of non-institutionalized or residents from geriatric care facilities, hospitals, or long-term nursing homes [7,8]. Specifically, older institutionalized individuals are experiencing a rapid decline of physical function often accompanied and partly caused by malnutrition and physical inactivity as soon as they change their living situation from “free-living” into an institutionalized surrounding [9,10].

Physiologically, the total energy intake decreases with age [11,12], which could result in concomitant declines in most nutrient intakes. Lower food intake among elderly people has been associated with lower intakes of e.g., iodine, calcium, iron, zinc, B vitamins, and vitamin E [13,14]. Several studies have demonstrated a remarkable impact of micronutrient status on health benefits and disease prevention in elderly people. The nutritional status has been identified as a major factor influencing immunity in this population [15].

Of particular interest in this respect are antioxidants, such as vitamin E or carotenoids that reduce lipid peroxidation and free radical damage. These nutrients have been linked to longevity [14,16] mainly due to their antioxidant and anti-inflammatory effects. There is evidence that these carotenoids strongly support health throughout the lifespan: A low intake of carotenoids has been associated with all-cause mortality, macular degeneration and associated blindness, cognitive decline, cardio-vascular diseases (CVD), various types of cancer, metabolic syndrome, oxidative damage to DNA, high blood pressure, hearing loss, decreased visual acuity, inflammation, and immune decay [16]. 

On the other side, consumption of antioxidants could potentially negatively influence optimal adaptation after exercise and should be considered carefully, especially in the elderly, where a low status of several micronutrients is common [17,18]. A sufficient availability of nutrients, especially antioxidant vitamins, such as vitamin C and E, is recommended to support antioxidant defense mechanisms. However, supplementing these nutrients, in too close proximity to an exercise stimulus, seems to restrain optimal adaptation of the redox-system [19,20].

Vitamin D is a key endogenous secosteroid hormone and nutrient, the major source of which is cutaneous synthesis following ultraviolet-B solar irradiation. Serum or plasma 25-hydroxyvitamin D (25(OH)D) concentration is the best index of vitamin D status as it reflects the sum of vitamin D synthesized in the skin and ingested in the diet. Circulating 25(OH)D concentrations are associated with skeletal and non-skeletal chronic diseases including osteoporosis, colon cancer, autoimmune diseases, cognitive state, and cardiovascular diseases in epidemiological studies [21,22]. A meta-analysis from a large consortium of cohort studies from Europe and the US demonstrated that comparing bottom versus top quintiles for 25(OH)D resulted in a pooled risk ratio of 1.57 (95 % CI 1.36 to 1.81) for all-cause mortality [23]; a result which was also confirmed in a recent systematic review of observational studies [24].

Since data on the status of fat soluble micronutrients are limited, particularly in subjects close to the 9th decade of life and also living in institutions, our aims of this secondary analysis of the ‘Vienna Active Ageing Study’, where we already reported on DNA and chromosomal damage, inflammation, oxidative stress, physical function, muscle quality, and sarcopenia [25,26,27,28,29], but so far not on the micronutrient status were first—to investigate and evaluate the plasma status of carotenoids, retinol, tocopherols, and vitamin D, in a cohort of institutionalized elderly and second—to assess the effects of a six-months lasting strength training with or without supplementing (antioxidant) vitamins and protein on the plasma status of six carotenoids, retinol, alpha(α)- and gamma(γ)-tocopherols and 25(OH)D. 

## 2. Materials and Methods

The presented data are part of the ‘Vienna Active Ageing Study’, which is a multidisciplinary project with partners from the Centre for Sport Science and University Sports, the Faculty of Life Sciences, the Research Platform Active Ageing (all, University of Vienna), the Karl Landsteiner Institute for Remobilization and Functional Health (Institute for Physical Medicine and Rehabilitation) and the Curatorship of Viennese Retirement Homes. The main characteristics and physical performance outcomes of this study have been already described previously [30].

### 2.1. Subjects

**Figure 1:**
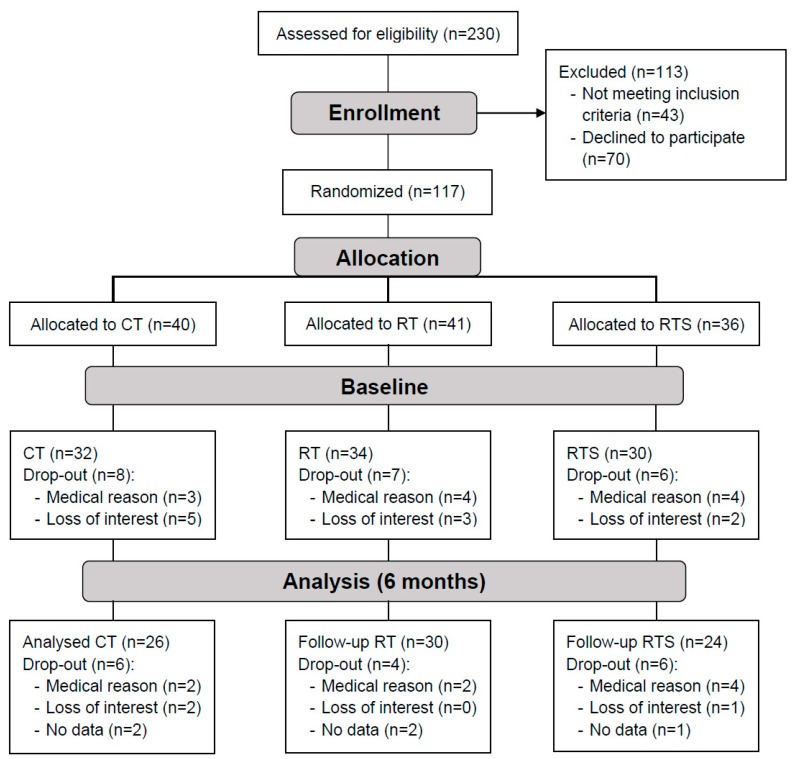
Participants flow diagram, Vienna Active Ageing Study.

One hundred and seventeen institutionalized elderly women and men (aged 65–98 years) were recruited from five different senior residencies in the area of Vienna (Curatorship of Viennese Retirement Homes) (Figure 1). The subjects were mentally (Mini Mental State Examination ≥ 23) and physically (Short Physical Performance Battery > 4) able to participate in this training intervention study. They were sedentary (less than one hour of physical activity or exercise per week) and free of severe diseases that would contra-indicate medical training therapy or measurement of physical performance, including cardiovascular diseases, diabetic retinopathy, and regular use of cortisone-containing drugs. Inclusion and exclusion criteria have been described in detail by Oesen et al. [30]. The health condition of all study subjects was assessed by specialists in internal medicine and gerontology. Written informed consent was obtained from all participants before entry into the study in accordance with the Declaration of Helsinki. Subjects were not allowed to take part in any exhausting physical activity within 2 days before the blood sampling and fitness test. All participants followed their medication protocols as prescribed by their physicians. If supplements were consumed before entering the study, details on further intake were discussed with their physicians.

### 2.2. Study Design

The present investigation is a secondary analysis and based on the study design which was described previously in Franzke et al. [31]. Briefly, study participants were randomly assigned into three intervention groups—cognitive training (CT), resistance training (RT), RT + supplement (RTS)—and stratified for gender in a randomized, controlled, observer-blind design. At baseline and after six months, blood samples were taken as well as physical and functional tests were performed. The study was conducted to investigate the effect of six months of elastic band resistance training, either with or without consuming a supplement containing macro- and micro-nutrients on markers of oxidative stress, antioxidant potential, oxidized DNA/RNA, functional parameters or the nutrient status in Austrian institutionalized elderly. The effects of either a resistance training intervention, a resistance training and nutritional supplementation intervention, or a cognitive training intervention (serving as a control group for the physical training groups) on markers of oxidative stress and antioxidant defense in institutionalized elderly, was assessed. The study was approved by the ethics committee of the City of Vienna (EK-11-151-0811) and registered at ClinicalTrials.gov, NCT01775111.

### 2.3. Resistance Training

The resistance training groups (RT and RTS) received two weekly sessions of resistance training, conducted on two non-consecutive days and were supervised by a sports scientist. Training attendance was recorded every session. Exercises were conducted using elastic bands, chairs, and own body weight—for detailed training program see supplement of Oesen et al. [30]. The progressive resistance training protocol was designed based on the guidelines of the American College of Sports Medicine for resistance training with older subjects [32]. The workout lasted for about one hour and consisted of an initial 10 min warm up, 30–40 min strength training for the main muscle groups (legs, back, abdomen, chest, shoulder and arms), and a 10 min cool down. The participants were motivated and controlled to adapt the resistance of the elastic band (shorter or stronger band) to keep exercise intensity within an effective range. After completing the initial phase of 4 weeks, where one set of 15 repetitions was performed, the intensity and volume progressively increased from two sets of light exercises to two sets of heavy resistance.

### 2.4. Resistance Training and Supplementation

The RTS group performed the same exercises together with the RT group and additionally received a liquid supplement every morning, as well as directly after each training session. Each drink supplied a total energy of 150 kcal and contained 20.7 g protein (56 energy (En)%, 19.7 g whey protein, 3.0 g leucine, >10 g essential amino acids), 9.3 g carbohydrates (25 En%, 0.8 BE), 3.0 g fat (18 En%), 1.2 g roughage (2 En%), 800IU (20 μg) of vitamin D, 250 mg of calcium, vitamins C, E, B6 and B12, folic acid, and magnesium (FortiFit, NUTRICIA GmbH, Vienna, Austria). The intake of the nutritional supplement was controlled at breakfast as well as after the training sessions.

### 2.5. Cognitive Training

The CT group served as our control group and performed coordinative or cognitive tasks [33] two times per week, equally to the frequency of the RT and RTS groups. This was done to minimize the “bias” of being part of a social group activity.

Participants of all groups were instructed to maintain their regular food intake, which was controlled by food diaries and 24 h recalls.

### 2.6. Measurements of Plasma Micronutrients

Blood samples were collected early morning after an overnight fast using heparin, serum and EDTA tubes (Greiner Bio-One, Kremsmunster, Upper Austria, Austria) and were processed immediately. Blood was centrifuged at 3000 rpm for 10 min to separate plasma from cells. The plasma for the micronutrients assessments was immediately frozen in aliquots at −80 °C until analysis. Plasma concentrations of retinol, α- and γ-tocopherol, α- and β-carotene, lutein, zeaxanthin, β-cryptoxanthin and lycopene were simultaneously measured by High Performance Liquid Chromatography (HPLC) with UV (retinol at 325 nm and the carotenoids at 450 nm), and fluorescence detection (tocopherols; Ex: 295 nm; Em:330 nm) (LaChrom Merck, Hitachi, Vienna, Austria) slightly modified as described earlier [34]. Briefly, the mobile phase was a 86:10:4 mixture of acetonitrile—methanol—2-propanol, the flow rate was 0.8·mL/min, the column temperature was set at 15 °C, and the column was a Aquasil RP-C18, 100 × 4 (5 µm particle size; Thermo Scientific, Vienna, Austria). After protein precipitation with ethanol, the micronutrients were extracted with hexane, which was then evaporated under nitrogen at 40 °C and reconstituted in the mobile phase. Tocol and ethyl beta-apo-8-carotenoat were used as internal standards. All CVs for the detected micronutrients were below 5%. Serum 25(OH)D levels were assessed by a direct competitive chemiluminescent immunoassay using the DiaSorin Liaison (DiaSorin, Saluggia, Piedmont, Italy) as described earlier [35]. 

### 2.7. Dietary Assessment

The intake of vitamins was assessed by interview-based 24 h recalls, which were performed at baseline and after 6 months. The evaluation of the records was performed using the nutritional software NUT.S (Dato Denkwerkzeuge, Vienna, Austria), which is based on the German Food Composition Database Version II.3 (Berlin, Germany) but was adapted for Austrian eating habits through the addition of typical Austrian recipes.

### 2.8. Chair Rise Test

In the 30-s chair rise test, where participants had to stand up and sit down from a chair (46 cm seat height) as often as possible within 30 s, was performed as previously described in Oesen et al. [30]. 

### 2.9. Handgrip Strength

To assess handgrip strength, participants performed an isometric handgrip strength test (kg) using a dynamometer. The test was conducted in a sitting position and maximal isometric contraction within 4–5 s was measured (JAMAR compatible handgrip dynamometer adapted to handle different sizes). The highest score of maximum voluntary contraction was used for data analyses [36].

### 2.10. Six-Minutes-Walking Test

Participants had to walk for six minutes as fast and as far as possible. The six-minutes-walking test is a valid tool to evaluate aerobic endurance in the elderly and was performed as previously described in Oesen et al. [30].

### 2.11. Isokinetic Dynamometry

Isokinetic peak torque measurements of knee extensors and flexors were performed using a LIDO Multijoint II isokinetic loading dynamometer (Loredan Biomedical Inc., Sacramento, CA, USA), as previously described in Oesen et al. [30]. 

### 2.12. Statistics

Statistical analyses were performed with IBM SPSS 24 (IBM SPSS Statistics for Windows, version 24.0, IBM Corporation, Armonk, NY, USA). For all parameters included into the current analyses, the Shapiro–Wilk test was used to check for normal distribution. Differences between gender and age-groups were measured using Kruskall–Wallis H and/or Mann–Whitney U Test. To assess the overall intra-group differences between the time points, dependent T test or Wilcoxon test (not normally distributed data) were conducted. Baseline and 6 months intervention differences as well as baseline changes between intervention groups were determined using a repeated measures analysis of variance.

Linear correlations were calculated using the Spearman test. A *p*-value of less than 0.05 was considered significant. 

## 3. Results

### 3.1. Baseline Characteristics of Fat Soluble Micronutrients

At baseline, 96 participants completed all tests (Figure 1). The ratio of 12.4% male and 87.6% female presents a representative gender distribution in the houses of the Curatorship of Viennese Retirement Homes. The mean age of the participants was 83.1 ± 6.1 years, 82.9 ± 6.0 years for women and 84.9 ± 6.7 years for men. Neither of the vitamin status did differ between RT, RTS or CT groups at baseline (RT vs. RTS vs. CT: Vitamin D, *p* = 0.338; retinol, *p* = 0.095; lutein, *p* = 0.649; zeaxanthin, *p* = 0.219; lycopene, *p* = 0.369; ß-cryptoxanthin, *p* = 0.076, α-carotene, *p* = 0.266; β-carotene, *p* = 0.106, α-tocopherol, *p* = 0.882; γ-tocopherol, *p* = 0.409). 

At baseline we observed significant correlations between lycopene and the peak torque knee extension (r = 0.366, *p* = 0.001; the 6-min-walking test (r = 0.241, *p* = 0.025) and the body weight (r = 0.217, *p* = 0.043). β-carotene correlated with the 6-min-walking test (r = 0.239, *p* = 0.026) and the body weight (r = 0.218, *p* = 0.042). The waist circumference correlated negatively with the baseline concentration of zeaxanthin (r = −0.312, *p* = 0.004), lycopene (r = −0.231, *p* = 0.030) and β-carotene (r = −0.261, *p* = 0.014). The hip circumference correlated negatively with the baseline concentration of zeaxanthin (r = −0.312, *p* = 0.004) and β-carotene (r = −0.288, *p* = 0.007).

### 3.2. Plasma Status of Fat Soluble Micronutrients of Institutionalized Elderly

Baseline data of the measured fat soluble micronutrients compared to reference values are presented in Table 1. Retinol status was very satisfactory with all subjects at or above the recommended level, whereas almost 3 out of 4 subjects had a β-carotene deficiency in plasma. The 25(OH)D status showed a similar pattern, where, depending on the reference threshold of 50 or 75 nmol/L, 61% or 81% of the investigated population experienced a deficit.

Sixty-seven % of the elderly showed satisfying plasma levels of α-tocopherol. All carotenoids without a reference value were in the range presented in other studies of younger populations, showing a broad range from being not detectable up to high plasma levels. 

There was no gender difference in the fat soluble micronutrient status. Furthermore, there was no statistical difference in micronutrient plasma status when comparing the age groups 65 to 74 years (11% of study population), 75 to 84 years (39%) and 85 and older (50%); data and analysis not shown.

The daily intake data were 1.2 mg for retinol (recommendation: >1 mg/d), 9.8 mg for α-tocopherol (recommendation: >12 mg/d), and 4.45 µg for vitamin D (recommendation: >20 µg/d) [37].

### 3.3. Intervention

Overall attendance in the training sessions was 71% (±26.5%) with no significant differences between groups (RT: 74.5% ± 20.8%, RTS: 70.7% ± 25.5%, 68.7% ± 32.5%; *p* ≥ 0.05). After six months of intervention, we observed significant improvements in both exercise groups in the chair rise (RT: *p* = 0.006, RTS: *p* = 0.002) and six-minutes-walking test (RT: *p* = 0.004, RTS: *p* = 0.029). There were no changes in the CT group. For further details about the effects on physical function parameters please see Oesen et al. [30]. There was no BMI change across the study.

The impact of the intervention on fat soluble micronutrients is shown in Table 2. There were only moderate modifications in the status of fat soluble micronutrients. In both the CT and RTS groups there were no significant changes after six months. In the RT group retinol, as well as lycopene, status increased significantly. This could be due to seasonal intake of foods containing these micronutrients. Between group evaluations showed no differences at baseline, not after 6 months and also not when considering changes (Δ) within groups.

The percentage of subjects meeting the recommended plasma levels (see Table 1) after the 6 months was for retinol still 100%, for β-carotene only 23%, and for α-tocopherol 67%, which was in the same range as baseline data. For vitamin D the percentage of subjects reaching 50 and 75 nmol/L increased to 51% and 26%, respectively.

After six months, daily intake data for retinol (RT/RTS/CT: 1.1 ± 0.6/1.3 ± 0.5/1.1 ± 0.5 mg) and for α-tocopherol (RT/RTS/CT: 9.3 ± 4.6/12.4 ± 7.8/8.8 ± 5.3 mg) showed no differences between groups. For vitamin D however, there was a significant increase (*p* < 0.05) in the daily intake in the RTS group (20.4 ± 4.4 µg) compared to the RT and the CT group (RT/CT: 5.2 ± 3.4/4.2 ± 3.3 µg).

### 3.4. Correlations after Six Months of Intervention

After six months some correlations could be assessed, however, there was no consistency for a specific nutrient to be correlated with outcome markers of physical activity.

**Figure 2:**
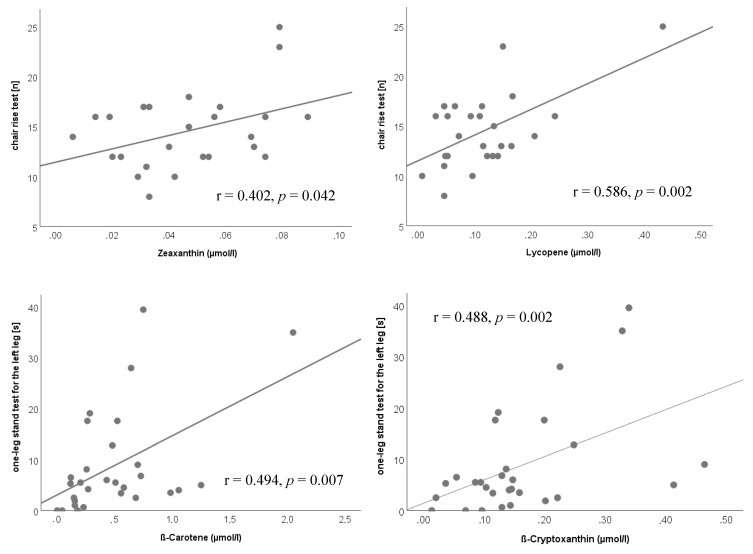
Correlations after 6 months of intervention in the resistance trained (RT) group.

In the RT group, the chair rise test correlated with the plasma levels of zeaxanthin (r = 0.402, *p* = 0.042) and lycopene (r = 0.586, *p* = 0.002). Further, the one-leg stand test for the left leg was correlated to β-cryptoxanthin (r = 0.488, *p* = 0.002) and β-carotene (r = 0.494, *p* = 0.007) plasma status (Figure 2). 

For the RTS group, no correlation could be observed between the micronutrients’ plasma status and physical outcome parameter.

**Figure 3:**
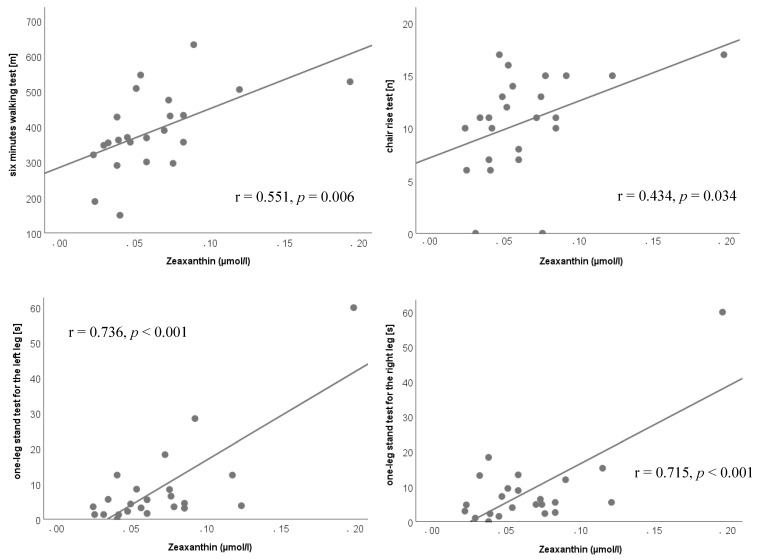
Correlations after 6 months of intervention in the control (CT) group.

In the CT group the plasma status of zeaxanthin showed positive correlations with the six-minutes-walking test (r = 0.551, *p* = 0.006), the chair rise test (r = 0.434, *p* = 0.034) and the one-leg-stand test (right leg: r = 0.715, *p* < 0.001; left leg: r = 0.763, *p* < 0.001); however a negative correlation with the six meter walking speed (r = −0.600, *p* = 0.002) occurred (Figure 3). 

Interestingly, there was no single correlation of physical fitness outcome parameter with vitamin D and no correlation between handgrip strength with the measured micronutrients.

When correlating changes (Δ) in plasma levels with changes in performance outcome parameter, the following links were observed: 

RT group: Δ Isokinetic dynamometry is correlating with Δ plasma retinol (r = 0.374, *p* = 0.024), lutein (r = 0.409, *p* = 0.013), zeaxanthin (r = 0.400, *p* = 0.016) and α-tocopherol (r = 0.383, *p* = 0.021); Δ handgrip is correlating with Δ plasma lycopene (r = 0.460, *p* = 0.005), α-carotene (r = 0.434, *p* = 0.008) and β-carotene (r = 0.373, *p* = 0.025); Δ six-minutes-walking test is correlating with Δ plasma lutein (r = 0.415, *p* = 0.012), lycopene (r = 0.481, *p* = 0.003) and β-carotene (r = 0.374, *p* = 0.025).

RTS group: Δ Isokinetic dynamometry is correlating with Δ plasma α-carotene (r = 0.363, *p* = 0.049); Δ handgrip is correlating with Δ plasma lycopene (r = 0.419, *p* = 0.021); Δ six-meter walking speed is correlating with Δ plasma retinol (r = 0.586, *p* = 0.001).

CT group: Δ one-leg stand test for the left leg is correlating with Δ plasma β-carotene (r = 0.532, *p* = 0.001), retinol (r = 0.445, *p* = 0.008).

## 4. Discussion

This secondary evaluation of the Vienna Active Ageing Study was conducted to evaluate the plasma status of ten fat soluble micronutrients in a cohort of institutionalized elderly Viennese women and men, and to further investigate the effect of six months of either strength training, strength training and a protein-vitamin supplement, or a cognitive intervention on their plasma status.

In various publications, fat soluble micronutrients have been linked to healthy aging and longevity. Among the top ten “longevity compounds”, the carotenoids lutein, zeaxanthin, lycopene, α- and β-carotene, β-cryptoxanthin, and astaxanthin have been highlighted very recently by Bruce Ames [16]. Vitamin E is linked to aging primarily due to its antioxidative effect and ability to delay the generation of reactive oxygen species, which are generally increasing within the aging process [14]. 

Regarding micronutrients in respect to aging, a vast part of the literature focused on 25(OH) D (e.g., [41]), as hypo-vitaminosis D is a condition highly prevalent worldwide and in particular in older adults [42].

Interventional studies of 25(OH) D supplementation have yielded positive effects e.g., on frailty status, mainly via improvements in physical performances [41]. Vitamin D3 supplementation in humans has been shown to positively influence musculoskeletal health in older adults by increasing the relative number as well as the cross-sectional area of muscle fibers (type II in particular), by increasing muscle strength and further by decreased fall rates [43,44,45].

### 4.1. Plasma Status of Fat Soluble Micronutrients of Institutionalized Elderly

The plasma status as well the intake of retinol in the study participants was very satisfying. 

For vitamin E, which is not only one of the major antioxidants but also exerts anti-inflammatory effects, the intake was lower than recommended and the plasma status could be improved. Every third participant showed an in-sufficient vitamin E status of less than 11.6 µmol/L. This is in the same range, although a little bit higher, compared to a recent study in Spain where institutionalized elderly around the same mean age, suffering from Chronic Obstructive Pulmonary Disease (COPD), were investigated on their plasma antioxidant status [46]; in this study, approximately every fourth participant showed a plasma status below 11.6 µmol/L.

The mean α-tocopherol levels were also lower compared to a recent European study where plasma carotenoids, tocopherols, and retinol were assessed in healthy participants aged 35–74 years [38]. However, the oldest participants of the latter study were in average around one decade younger than the subjects investigated in this study. Since a sufficient vitamin E status as well as an appropriate vitamin E intake above 12 mg/d have been shown to be important to avoid detrimental effects e.g., on bone, muscle mass, and cognitive function [47], vitamin E rich food such as oat meals, nuts, almonds, or vegetable oils e.g., for salad, should be provided for this very specific population. One limitation in increasing micronutrient intake is the decreasing total energy intake in this later period of life. We assessed an average daily energy intake of around 1550 kcal (Baseline: 1514 kcal/d; after intervention: 1572 kcal/d; no significant differences), which points to the importance of using nutrient dense food in order to maintain sufficient micronutrient levels. Otherwise, if it is not possible to achieve an appropriate plasma status, the use of supplements should be considered to avoid symptoms of deficiency.

Reference values are largely not available for plasma carotenoids. For β-carotene, 0.75 µmol/L is discussed [48], a threshold which exceeded only 27% of the participants. This is even higher compared to data of the Austrian Nutrition Report 2012, where only 11% exceeded this threshold level [39]. The mean value of 0.56 ± 0.19 µmol/L is comparable to a recent study, which combined data of four European cohorts of older adults with a mean age of 77.6 years [49]. In this study the plasma β-carotene level was 0.44 ± 0.51 µmol/L, in the Spanish COPD study 0.38 ± 0.42 µmol/L [46].

The same can be observed for other carotenoids, where the mean data are also in line with the mentioned studies [38,46,49]. A large inter-individual range within our study group e.g., for lutein from 0.04 µmol/L up to 2.03 µmol/L, was observed. This might be attributed to different fruit and vegetable intakes, which is however generally very low in this population group. For fruits, the recommendation is only met by 39% of Austrian elderly, for vegetables and pulses only by 28% [39], which also explains the broad range in the plasma levels.

The association of vitamin D levels with bone health and fracture risk has often been described in older adults [50,51]. Various studies showed a high percentage of vitamin D deficiency in this population [52] with around 60% to 93% of subjects having vitamin D plasma levels below 50 nmol/L [53]. Our data are in line with these observations with only 39% or 19% of the elderly above 50 nmol/L or 75 nmol/L plasma levels, respectively. Intake data revealed also an inadequate supply with vitamin D. 

Since vitamin D insufficiency causes—or has been associated with—a large number of diseases that affect healthy aging, such as all-cause mortality, cancer, CVD, diabetes, or brain function [16], moderate dose vitamin D supplementation (for reaching 25(OH) D plasma levels between 50 and 100 nmol/L) in this high age group is recommended. 

### 4.2. Effect of the Intervention on Plasma Status of Fat Soluble Micronutrients of Institutionalized Elderly

The effect of six months training intervention with or without protein-vitamin supplementation was very moderate on the status of fat soluble micronutrients. In the CT group there was no significant change in the micronutrient intake as well as the plasma status. The same is true for the RTS group despite the intake of vitamin D, which increased significantly from 5.89 µg/d at baseline to 20.4 µg/d after six months. This can be explained by the supplement intake, which contained 20 µg vitamin D per portion (additional 180 µg/week). This promoted the increase in the plasma levels by 11%, although, this increase was not significant, but it contributed to a slight improvement of subjects exceeding 50 nmol/L (39% at baseline to 51% in the RTS group after intervention). Although vitamin A and E was part of the supplement in low amounts, this had no effect on the plasma levels after 6 months.

In the RT group there was a significant increase in retinol and lycopene status, however, the absolute levels only changed moderately with no expected biological effects. 

This indicates no additional production of free radicals due to the training stimulus over 6 months, which might have influenced the antioxidant status. 

Performance parameters were not linked to any improvements in fat soluble micronutrient plasma levels. The observed correlations mainly with zeaxanthin, lycopene, β-carotene, or β-cryptoxanthin, are interesting and sometimes strong, however, following no consistency and were mainly seen in the CT group and the RT group. Particularly in the RT group, several correlations were observed between the changes in micronutrient status and changes in performance parameters. However, it points to the importance of an appropriate carotenoid intake. Interestingly, there was no single link between vitamin D status and performance parameters, which was also true for the RTS group, despite receiving vitamin D with the supplement. 

Noteworthy, this study has some limitations. Although our in- and exclusion criteria aimed at getting a very homogenous sample of elderly people, the very advanced age of our participants (around or above their statistical live expectancy) led to a broad standard deviation in most of our parameters by nature. On the other hand, this is one of the strengths of the study since micronutrient status and intake data are scarce in this very old age group and data are reflecting the broadness of this age group. This is also mirrored in the high number of female participants in this study, as the average age in our study population was around four years older than the average life expectancy for males. Dietary assessment was also sometimes not easy in this age group and needed specific training of the interviewer, took longer than regularly for adults, and we cannot guarantee some missing food items, although there were often check backs with the kitchen and/or the care personnel of the facilities. 

## 5. Conclusions

In this study cohort of old to very old institutionalized elderly, we could observe a diverse plasma status of fat soluble micronutrients; appropriate for retinol, improvable for α-tocopherol, and not satisfying for β-carotene, and particularly vitamin D. Plasma status of other carotenoids showed a broad range, from being not detectable up to quite high values, reflecting a diverse intake of fruits and vegetables in this age group.

Six months of elastic band resistance training with or without protein-vitamin supplementation improved physical function, but had no biological impact on the status on fat soluble micronutrients. The findings in our study encourage to put a stronger focus on the supply of nutrient dense plant food—such as eating a colorful variety of vegetables and fruits (“eating the rainbow”), cereals, and plant oils every day added with a frequent consumption of nuts and fatty fish—in this age group in order to obtain sufficient micronutrient levels or, if not otherwise possible such as for vitamin D, to use specific supplements. 

## Figures and Tables

**Table 1:** Status of fat soluble micronutrients of institutionalized elderly at study entry compared to reference values [38,39,40].

Parameter	Plasma Status at Study Entry	Reference Plasma Value	% of Deficient Participants
Subjects [number]	96		
Age [years]	83.1 (65–98)		
Retinol [µmol/L]	2.20 (1.05–4.16)	>1.05	0%
Lutein [µmol/L]	0.25 (0.04–2.03)		
Zeaxanthin [µmol/L]	0.040 (n.d.–0.19)		
Lycopene [µmol/L]	0.009 (n.d.–0.37)		
β-Cryptoxanthin [µmol/L]	0.18 (n.d.–0.80)		
α-Carotene [µmol/L]	0.05 (n.d.–0.46)		
β-Carotene [µmol/L]	0.42 (n.d.–2.4)	>0.75	73%
α-Tocopherol [µmol/L]	16.51 (n.d.–26.8)	>11.6	33%
γ-Tocopherol [µmol/L]	2.63 (1.80–9.14)		
Vitamin D [nmol/L]	44.0 (9.0–162)	>50/75	61%/81%
Data are median (Min–Max), n.d. not detectable			

**Table 2:** The impact of six months (6 Mo) of intervention on the status of fat soluble micronutrients.

	RT		RTS		CT	
	Baseline	6 Mo	Baseline	6 Mo	Baseline	6 Mo
Subjects [number]	34	30	30	24	32	26
BMI [kg/m^2^]	29.0 ± 3.7	29.0 ± 3.9	30.0 ± 6.3	30.0 ± 5.4	28.6 ± 4.9	27.9 ± 5.3
Retinol [µmol/L]	2.14 ± 0.50	2.31 ± 0.49 *	2.15 ± 0.49	2.28 ± 0.66	2.46 ± 0.67	2.47 ± 0.53
Lutein [µmol/L]	0.31 ± 0.34	0.28 ± 0.23	0.33 ± 0.22	0.28 ± 0.19	0.40 ± 0.39	0.50 ± 1.09
Zeaxanthin [µmol/L]	0.04 ± 0.02	0.05 ± 0.03	0.05 ± 0.03	0.05 ± 0.03	0.06 ± 0.04	0.07 ± 0.06
Lycopene [µmol/L]	0.09 ± 0.08	0.10 ± 0.08 *	0.11 ± 0.07	0.10 ± 0.07	0.12 ± 0.07	0.11 ± 0.08
β-Cryptoxanthin [µmol/L]	0.16 ± 0.10	0.16 ± 0.11	0.23 ± 0.13	0.18 ± 0.15	0.24 ± 0.18	0.21 ± 0.14
α-Carotene [µmol/L]	0.05 ± 0.10	0.09 ± 0.14	0.10 ± 0.12	0.12 ± 0.11	0.08 ± 0.07	0.12 ± 0.12
β-Carotene [µmol/L]	0.41 ± 0.44	0.49 ± 0.43	0.61 ± 0.45	0.55 ± 0.51	0.68 ± 0.51	0.67 ± 0.46
α-Tocopherol [µmol/L]	19.94 ± 13.96	21.03 ± 14.62	21.47 ± 17.04	21.17 ± 19.61	19.48 ± 14.50	19.73 ± 15.02
γ-Tocopherol [µmol/L]	4.81 ± 5.84	4.72 ± 4.71	3.26 ± 3.07	2.84 ± 2.54	3.52 ± 3.33	3.59 ± 3.43
Vitamin D [µmol/L]	54.12 ± 27.6	55.98 ± 25.56	59.35 ± 41.48	66.33 ± 35.04	46.9 ± 31.02	53.04 ± 25.13

Data are means ± SD; *p*-values are calculated using paired *t* test or Wilcoxon test between time points, * *p* < 0.05.
